# Challenge Test Analysis of *Salmonella* Behavior During Sardinian Fermented Sausage Production and Storage †

**DOI:** 10.3390/foods15060986

**Published:** 2026-03-11

**Authors:** Giuliana Siddi, Francesca Piras, Maria Pina Meloni, Mattia Migoni, Mario Cuccu, Myriam Casula, Fabiana Manca, Fabrizio Simbula, Enrico Pietro Luigi De Santis, Christian Scarano

**Affiliations:** Department of Veterinary Medicine, University of Sassari, Via Vienna, 2, 07100 Sassari, Italy; g.siddi1@phd.uniss.it (G.S.); mariapinameloni@yahoo.it (M.P.M.); mattiamigoni35@gmail.com (M.M.); mrcuccu@uniss.it (M.C.); myriamcasula@alice.it (M.C.); fabianamanca36@gmail.com (F.M.); simbula.fabrizio@gmail.com (F.S.); desantis@uniss.it (E.P.L.D.S.); scarano@uniss.it (C.S.)

**Keywords:** pork, fermented meat product, shelf life, ripening

## Abstract

This study evaluated *Salmonella* behavior during Sardinian fermented sausage (SFS) production through a challenge test on experimentally inoculated raw meat. The objectives were to (i) determine the survival and reduction kinetics of *Salmonella* during fermentation and ripening and (ii) evaluate the relationship between pathogen behavior and the evolution of key chemical-physical parameters (pH, water activity). Three batches of SFS were produced, and the meat mixture was inoculated with a three-strain *Salmonella* cocktail (reference and field strains) to 10^2^ CFU/g. After 20 days of ripening, sausages were vacuum-packed and stored under refrigerated conditions (+4 ± 2 °C). For each batch, triplicate samples were collected and analyzed at different production stages (mixing, after overnight rest, and 24 h after stuffing) and during shelf life (days 6, 21, 30, and 40). Analyses included *Salmonella* detection and enumeration by direct plating, aerobic colony count, *Enterobacteriaceae*, staphylococci, lactic acid bacteria, molds and yeasts, as well as pH, water activity, and gross composition. *Salmonella* counts increased by approximately one log unit after stuffing, before the onset of acidification. During fermentation and ripening, pathogen levels declined but remained detectable, even after prolonged refrigerated storage. These findings indicate that although ripening, and particularly fermentation, significantly (*p* < 0.05) reduce *Salmonella* levels, complete inactivation is not achieved. The study highlights the importance of controlling initial contamination levels, validating fermentation and ripening conditions, and the application of additional post-process hurdles to ensure product safety.

## 1. Introduction

“Salsiccia Sarda,” or Sardinian fermented sausage (SFS), is a traditional ready-to-eat (RTE) pork meat product typical of Sardinia (Italy) and included in the National List of traditional food products [1]. It is a Mediterranean-style, dry-fermented sausage made of minced pork meat and fat [2]. The production process involves the selection, chopping, and mincing of pork meat and fat, followed by mixing with curing ingredients, spices, and authorized additives, including nitrates and nitrites [3]. After overnight refrigerated storage, the mixture is stuffed in a bowel. The fermentation stage continues during the next steps of initial dipping (20–22 °C for 24 h) and drying (2–3 days). Ripening is carried out for about 20 days in storerooms at 15 °C [4,5].

SFS is produced throughout the whole region of Sardinia in numerous and different production realities, ranging from small and artisanal establishments to larger plants with industrial processing. The production is still heavily influenced by customs and family recipes, resulting in a high degree of variability in the manufacturing process [5]. For this reason, SFS’s safety is dependent on the application of multiple parameters as well as physicochemical conditions and “hurdles” at various phases of the fermentation and ripening process. A production process conducted in a targeted and correct way can guarantee proper acidification and drying, which result in final products with pH values ranging between 5.3 and 5.5 and a_w_ values ≤ 0.920 [5,6]. These hurdles, mainly established during drying and ripening, are critical in limiting *Salmonella* survival and supporting compliance with the qualitative microbiological EU criterion for *Salmonella* (absence in 25 g) [7].

Although traditionally considered low-risk products [8], fermented sausages are increasingly gaining attention due to their frequent involvement in recalls and outbreaks in Europe and the US, often linked to the presence of *Salmonella* [9,10,11,12,13,14]. Particularly, SFS is characterized by a short ripeningperiod and lacks microbicidal steps, which may allow pathogen survival. The risk of *Salmonella* is supported by market recalls of SFS, including a recent national recall [15] involving an SFS, as well as a European notification [16] concerning *Salmonella* in pork sausage. The detection of *Salmonella* both at the end of acidification and at the end of ripening is also consistent with previous findings from SFS facilities, where 24% of products after acidification and 2% after ripening tested positive [6].

The Challenge test is a tool designed to simulate the behavior and distribution of pathogens in food during production, under expected storage conditions, and during handling [17]. It involves a laboratory investigation in which the food is artificially contaminated with a known concentration of a specific pathogenic microorganism. The objectives are to assess the effectiveness of the production process in eliminating pathogens and to evaluate whether the ready-to-eat food provides conditions that support their growth [18]. However, the findings of these studies are specific to the food product and storage conditions tested, meaning the results of a growth potential study cannot be generalized to other products or storage conditions [19]. In this regard, previous studies have examined the survival of pathogens like *Listeria monocytogenes* in SFS [20]. However, there is limited evidence in the literature on how the production process impacts the initial contamination of meat with enteric pathogens such as *Salmonella*. In this framework, this study aimed to evaluate the influence of the production process and the final chemical-physical properties of SFS in controlling *Salmonella*, providing data to support the management of “*Salmonella* risk” within the production facility.

## 2. Materials and Methods

The protocol for the challenge test was defined according to [21]. The test was performed in a pilot plant located at the Department of Veterinary Medicine (University of Sassari) on three different batches of SFS, with experimental production of two types of samples per batch: (1) samples produced from minced meat contaminated with *Salmonella* strains during the mixing phase and (2) samples produced from meats without inoculation of *Salmonella* strains (control samples).

### 2.1. Inoculum Preparation

A total of three strains of *Salmonella* were used to create the pathogen inoculum, consisting of one reference strain and two field strains. The strains selected for inoculation were the following: *Salmonella enterica* serovar Braenderup H9812 strain (ATCC BAA-664), which accounted for the reference strain, *S.* Typhimurium monophasic variant (ST34), and *S.* Goldcoast (ST358), both isolated from pigs. The inoculum was prepared according to [20]. Briefly, the strains, stored frozen (−80 °C) in Brain Heart Infusion broth (BHI; Biolife, Milan, Italy) supplemented with 20% glycerol, were transferred (1% inoculum) to BHI and incubated at 37 °C for 24 h in aerobic conditions to ensure reaching the stationary phase. To acclimate the cells to the expected environmental conditions for the challenge test, the bacterial suspensions were stored at +4 °C for 7 days. The initial concentration of vegetative cells in the culture was 10^8^ CFU/mL. Serial decimal dilutions were prepared, and counts were confirmed by direct inoculation on Xylose Lysine Desoxycholate (XLD) agar (Microbiol, Cagliari, Italy) plates. Before use, the individual strains were combined in equal volumes in order to obtain a multi-strain cocktail and inoculated into the sausage mix to achieve a concentration of 10^2^ CFU/g. The artificial inoculation performed in this study was intended solely for challenge test purposes, not to assess regulatory compliance of experimentally inoculated products, and does not reflect the microbiological status of commercial products. This inoculum level was selected in accordance with [21], which recommends using contamination levels representative of realistic conditions and sufficiently low to avoid altering the natural microbial ecosystem and fermentation process while still allowing reliable pathogen enumeration. This inoculation level is also consistent with values commonly used in challenge tests on fermented meat products to evaluate process effectiveness under conditions representative of industrial production [22,23].

### 2.2. SFS Production

In the pilot producing plant, n. 3 batches of SFSs were produced. Two types of samples for each batch were produced: (1) negative control samples (C); (2) inoculated treatment group, including samples inoculated with *Salmonella* broth culture during the mixing step.

SFSs were manufactured according to the technological process applied by producing plants representative of the sector [5]. Briefly, the production process involved the selection, chopping, and mincing of pork meat and fat, followed by mixing with curing ingredients, spices, and authorized additives, including nitrates and nitrites. In this regard, at the time the experiments were conducted, the maximum permitted level of nitrites in meat products was 150 mg/kg, in accordance with [3]. This limit was subsequently revised to 90 and 80 mg/kg, applicable from 9 October 2025. The present study was performed in compliance with the regulatory limits in force at the time of production. A commercial starter culture consisting of lactic acid bacteria (LAB) and nitrate-reducing coagulase-negative staphylococci (CNS) was added during the mixing step according to the manufacturer’s instructions. In detail, the composition of the starter culture included *Latilactobacillus curvatus*, *Latilactobacillus sakei*, *Staphylococcus xylosus*, *Staphylococcus carnosus*, and *Mammaliicoccus vitulinus*. According to the manufacturer’s instructions, the starter culture was applied at a rate of 0.1 g/kg of meat mixture.

**Figure 1:**
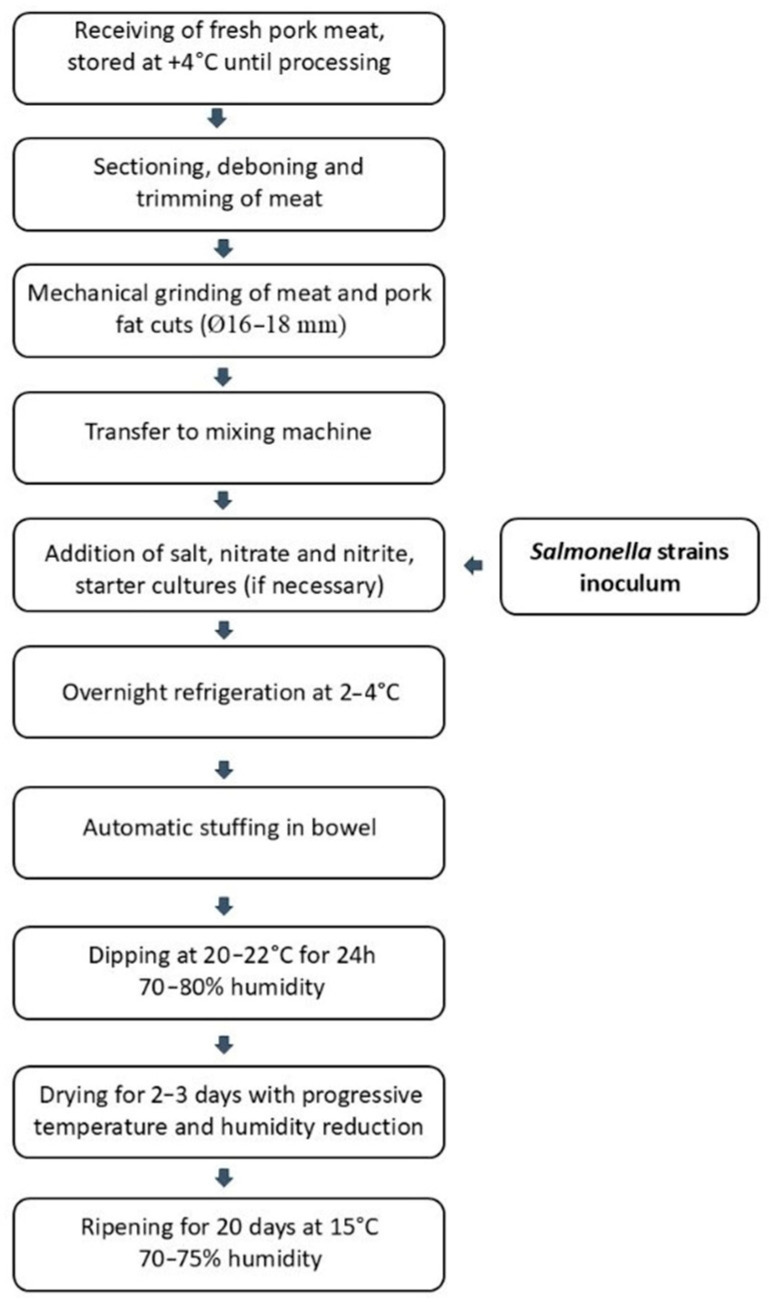
The production process of artificially contaminated SFS.

In the inoculated treatment group, the inoculum with *Salmonella* broth culture was added during the mixing step. After overnight refrigerated storage, the mixture was stuffed in natural bowel. The fermentation stage continued during the next steps of initial dipping (20–22 °C for 24 h) and drying (2–3 days). Ripening was carried out for 20 days in the storerooms at 15 °C. The production process of contaminated SFS is summarized in Figure 1.

In order to account for the typical techniques applied in industrial production plants, after 20 days of ripening, SFSs were vacuum-packed and stored at refrigeration temperature (+4 ± 1 °C). Each SFS was regarded as a sample.

Triplicate samples of each of the three batches of SFS were analyzed during the production process and during the shelf life of the vacuum-packed product at the following analysis times:

- minced meat at the end of the mixing phase (T_0_),
- minced mixture before the stuffing phase, following an overnight rest phase (T_1_),
- 24 h after stuffing (T_2_),
- 6 days after stuffing, at the end of the acidification (T_7_),
- 21 days after stuffing, at the end of ripening (T_21_),
- 30 days after stuffing, after refrigeration storage (T_30_),
- 40 days after stuffing, after refrigeration storage (T_40_).

Since, as it will be discussed further, the pathogen was present after 40 days, further analysis times were added until T_100_ (100 days after stuffing and after refrigeration storage).

### 2.3. Physico-Chemical and Composition Analysis

On each sample and at each time point, pH and water activity (a_w_) values were determined using a pH meter GLP 22 (Crison Instruments SA, Barcelona, Spain) and Aqualab CX3 (Decagon, Pullman, WA, USA). Moisture, fat, and protein (expressed as %) were determined by the FoodScanLab (FOSS Analytic, Hillerød, Denmark) using the Near-Infrared Transmittance (NIT) technology and a previously set calibration curve. Analyses were performed in triplicate on a homogenized sample representative of the product.

### 2.4. Microbiological Profile

At each time-point, *Salmonella* qualitative detection [24] was carried out on both control and treated samples. *Salmonella* count was performed by serial dilution and direct surface plating onto XLD agar. Samples in which *Salmonella* was not detected by the enumeration method were reported as below the detection limit (<1.0 log CFU/g). Moreover, the evaluation of the microbiological profile of the samples included the determination of the aerobic colony count [25], *Enterobacteriaceae* count [26], coagulase-positive and coagulase-negative staphylococci [27], mesophilic lactic acid bacteria [28], molds, and yeasts [29].

### 2.5. Pulsed-Field Gel Electrophoresis

To confirm the genetic relatedness between *Salmonella* isolates recovered during the challenge test and the strains included in the inoculum cocktail, pulsed-field gel electrophoresis (PFGE) was performed. PFGE analysis was performed using the ECDC standardized protocol [30]. PFGE analysis was performed on representative *Salmonella* isolates recovered at each sampling time throughout production and extended shelf life, including early, intermediate, and late stages of storage. At each sampling time, a minimum of two *Salmonella* isolates per sample were randomly selected to ensure representativeness. Overall, a total of 16 isolates recovered during the challenge test were subjected to PFGE and compared with the strains included in the inoculum cocktail. The comparison was performed by BioNumerics software v7.1 (Applied Maths, Saint-Martens-Platen, Belgium). Cluster analysis was performed by the Dice similarity coefficient, with 1% optimization and 5% tolerance using the unweighted pair group method with arithmetic mean (UPGMA).

### 2.6. Statistical Analysis

Differences among pH, a_w_, and microbiological counts (log CFU/g) between control and inoculated samples at every time point were compared using the analysis of variance (ANOVA) model with post hoc Tukey HSD test for comparing multiple treatments. *Salmonella* counts (log CFU/g) were analyzed using a linear mixed-effects model, with sampling time as a fixed effect and batch as a random effect, to account for repeated measurements within batches. Values below the limit of detection (LOD = 1.0 log CFU/g) were treated as censored and replaced with LOD/2 for quantitative analysis. Post hoc pairwise comparisons between time points were performed with appropriate adjustment for multiple comparisons. Statistical significance was set at *p* < 0.05. Statistical analyses were performed with Statgraphics Centurion XIX software Version 19 (Stat Point Technologies, Warrenton, VA, USA).

## 3. Results

All PFGE profiles obtained from *Salmonella* isolates recovered at different sampling times were indistinguishable from those of the inoculum strains, indicating that the recovered isolates were genetically consistent with the strains used for inoculation and that no evidence of contamination by unrelated *Salmonella* strains was observed during processing and storage.

As shown in Table 1 and Table 2, no significant differences in pH, a_w_, and microbiological counts were observed between control and *Salmonella*-inoculated samples. As for physico-chemical characteristics, in both sample types, the stuffing and acidification/drying phases resulted in a progressive reduction in pH and water activity, with the lowest values recorded after 7 days. During ripening, pH slightly increased, whereas a_w_ further decreased and remained stable at low levels throughout refrigerated storage.

A significant effect of sampling time on *Salmonella* levels was observed (*p* < 0.05). After inoculation, *Salmonella* counts showed a slight increase after stuffing, followed by a reduction during the acidification/drying phase (*p* < 0.05), with the lowest levels recorded after 7 days. At the end of ripening, *Salmonella* remained detectable, and similar concentrations were observed during refrigerated storage under vacuum, although a further decrease was noted at the end of the storage period. No statistically significant differences were observed between initial contamination levels and levels measured at the end of ripening. Enrichment [21] results reflected the positivity in XLD agar direct plating, and all samples were also positive after plating on MSRV agar. Since *Salmonella* remained detectable at T_40_, further analyses were carried out during extended refrigerated storage. After 100 days, mean *Salmonella* counts were markedly reduced. While direct enumeration on XLD agar frequently resulted in non-detectable levels after 40 days, the pathogen was consistently detected by enrichment.

Microbial populations exhibited trends consistent with the fermentation and ripening process. The variability observed in LAB counts at T_0_ reflects the dispersion among replicate samples immediately after starter culture addition, prior to microbial stabilization during fermentation, due to the physiological state of the starter cultures and the complex structure of the meat matrix, which can influence cell recovery and enumeration efficiency [31]. LAB showed a marked increase during fermentation and stabilized thereafter, whereas total aerobic counts, *Enterobacteriaceae,* and non-aureus staphylococci displayed more limited variations. Molds and yeasts increased during ripening. No significant differences were detected between control and contaminated samples for any microbial group.

The centesimal composition of the starting mixture and products is reported in Table 3.

Ripening affected the centesimal composition of SFSs. The progressive decrease in moisture was associated with a concentration of fat and proteins, which reached their highest values after ripening and remained stable during storage. Sodium chloride levels increased during processing and showed limited variations thereafter.

## 4. Discussion

The safety of fermented sausages relies on the combination of multiple intrinsic and extrinsic hurdles to control pathogenic and spoilage microorganisms, including acidification, reduction in water activity (a_w_), addition of salt and curing agents, competitive activity of starter cultures, and oxygen depletion during fermentation and ripening [32]. Specifically, in fermented sausages produced at temperatures above 20 °C, such as SFS, pH reduction is generally regarded as the primary hurdle limiting pathogen growth [33]. This effect is due to the lactic acid produced by the starter cultures, which are generally added during the mixing phase and expected to reach 10^8^–10^9^ CFU/g [34]. Water availability is another key factor for the growth and survival of pathogens in dry fermented sausages. In this type of product, moisture loss during ripening and water binding by salt and other components progressively reduce a_w_ to levels increasingly unfavorable for microbial growth [32].

In our study, pH and a_w_ values reached 5.27 ± 0.35 and 0.95 ± 0.01 after 7 days from bagging, and after ripening, pH values increased to 5.45 ± 0.18, and a_w_ dropped to 0.814 ± 0.02. These values are lower than those typically reported for commercially available SFS samples [5,6] and represent advanced fermentation and drying conditions, as they create particularly restrictive conditions for microbial survival. This progressive dehydration was also reflected in compositional changes, with proportional increases in fat and protein percentages due to moisture loss, as values were expressed on a wet weight basis. This effect reflects the concentration of solid components during ripening [35,36]. A further slight decrease in moisture was observed after vacuum packaging, which may reflect ongoing moisture redistribution and equilibration within the product matrix [37].

**Figure 2:**
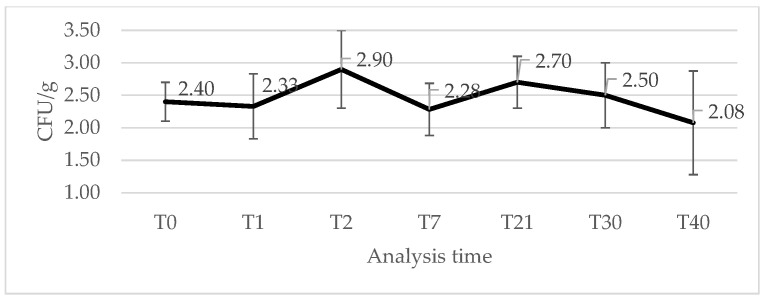
Mean *Salmonella* counts (log CFU/g) at each sampling time, with corresponding standard deviation error bars.

Despite the application of said hurdles, *Salmonella* was not completely eliminated. The temporal evolution of *Salmonella* levels (Figure 2) reflected the changing physicochemical conditions of the product.

After the inoculation and the overnight rest (T_1_), concentrations (2.33 ± 0.60 CFU/g) were relatively stable. During the very early stages of production, characterized by high pH and a_w_ and before stabilization of the fermentative microbiota, a slight increase in *Salmonella* counts was observed (T_2_: 2.90 ± 0.70 log CFU/g). This behavior is consistent with previous studies showing that early fermentation conditions may temporarily support pathogen growth [8,38,39,40]. However, this rise did not persist: by day 7, counts had decreased to 2.30 ± 0.80 log CFU/g, indicating a downward shift that corresponds with the concurrent pH reduction to 5.27 ± 0.35. Although this pH value does not fall within the no-growth range for *Salmonella* [41], acidification can impose sublethal stress and restrict its ability to multiply. Several studies demonstrate that *Salmonella* growth becomes progressively compromised as pH declines toward 5.3–5.0, with reduced growth rates and extended lag phases [38,42]. The observed reduction in viable counts is therefore consistent with the onset of acid-driven inhibition occurring, combined with increasing competition from lactic acid bacteria during the first week of ripening [8,40]. Together, these factors explain the downward trend observed at this stage of the process. A slight increase in *Salmonella* counts was observed between T_7_ and T_21_; however, this fluctuation is unlikely to reflect true bacterial proliferation. Rather, it could reflect the recovery of sub-lethally injured bacterial cells following the acute acid stress associated with fermentation [43]. At T_7_, the product is still at the end of the most intense drop in pH, a condition known to impose substantial acid stress on *Salmonella* and to reduce the culturability of cells on selective media [44]. Under such sublethal injury, a fraction of *Salmonella* cells may temporarily lose culturability on selective media while remaining viable [43,45,46]. The increase in pH observed during ripening may have reduced environmental stress and facilitated the recovery of sublethally injured cells, resulting in an apparent increase in CFU without actual population growth. Acidic conditions during fermentation can disrupt cellular homeostasis, including membrane integrity, enzyme activity, and intracellular pH regulation, resulting in sublethal injury and reduced culturability. Subsequent increases in pH may reduce environmental stress and allow cellular repair mechanisms to restore physiological functions and culturability [45]. Similar recovery phenomena following acid stress have been described in *Salmonella* and other *Enterobacteriaceae* in fermented and low-moisture foods [46,47]. Although this mechanism was not directly investigated in the present study, it represents a plausible explanation for the observed increase in counts during storage. This hypothesis is worth further targeted experimental approaches. Moreover, alternative explanations, including localized microenvironmental growth niches, cannot be excluded.

At the end of ripening, *Salmonella* levels (T_21_: 2.68 ± 0.40 log CFU/g) were close to those measured immediately after inoculation, indicating that while growth was effectively restricted, complete inactivation was not achieved. Subsequent vacuum packaging and refrigerated storage led to a gradual decline in counts, reflecting the additional stress imposed by low oxygen availability, low a_w_, and refrigeration. The relatively high variability of low *Salmonella* levels observed in samples during storage, highlighted by standard deviation values, is expected in challenge test studies, as small differences in bacterial counts translate into larger differences on a logarithmic scale [48]. The heterogeneous structure of fermented sausages may also result in localized differences in environmental conditions influencing pathogen survival [38].

As mentioned, *Salmonella* remained detectable throughout the shelf life and prolonged storage at 4 °C. The limited reduction observed during processing (approximately 0.4 log CFU/g), despite decreases in pH and a_w_, is consistent with the well-documented resistance of *Salmonella* in low-moisture fermented meat matrices [49,50]. Although acidification and drying represent important hurdles, pH values above 5.0 and low a_w_ primarily inhibit bacterial growth rather than ensuring rapid inactivation. While *Salmonella* cannot grow at a_w_ values below 0.930, it can persist for extended periods in low-moisture, high-fat foods, particularly under refrigeration [51,52,53]. In addition, exposure to sublethal stress during fermentation may induce adaptive responses that enhance pathogen tolerance [54].

The persistence of low-level contamination observed in this study is in line with previous investigations of SFS. A study on SFSs [6] reported *Salmonella* in finished products after ripening (2% prevalence). Another study on SFSs [5] found *Salmonella* in 2.8% of fully ripened SFS sampled across eight establishments.

Similar observations have been reported in both observational and challenge-test studies conducted on Italian fermented salami produced under comparable technological conditions. A study [55] examined 150 batches of dry-cured Italian salami manufactured from *Salmonella*-positive ground raw meat and found that, after ripening (20–86 days depending on the batch), approximately 13% of products remained *Salmonella*-positive. Other authors [8] inoculated *Salmonella* enterica into “Cacciatore” and “Felino” salami and reported very limited reductions during processing (approximately 1.1–1.6 log CFU/g), resulting in detectable levels of *Salmonella* at the end of the product. Additionally, challenge tests have been conducted on Italian salami from ten production facilities [56], monitoring time, temperature, pH, a_w_, and *Salmonella* counts throughout production. The authors showed that the production process alone does not always guarantee the commonly desired 5-log reduction, especially in salami with marginal hurdle conditions (higher pH, higher a_w_, shorter ripening), which supports the plausibility of detecting *Salmonella* in the final product under suboptimal conditions. The presence of *Salmonella* along the production chain of fermented sausages and in finished products has also been reported in studies conducted in Spain and France [57].

Overall, the data consistently show that while processing steps markedly limit pathogen growth, they do not ensure complete inactivation. Rather than indicating a limitation of fermentation as a control measure, these findings highlight that its effectiveness depends on the interaction of multiple intrinsic and extrinsic factors, including initial contamination levels, product composition, and processing conditions [33]. Fermentation and ripeningshould be regarded as important hurdles that contribute to pathogen control through acidification, reduction in a_w_, and microbial competition. However, these factors typically result in variable log reductions rather than complete pathogen elimination, with effectiveness influenced by initial contamination levels and technological conditions. Similar considerations are supported by documented *Salmonella* outbreaks and recalls associated with fermented meat products [15,16]. Consequently, the microbiological quality of raw materials represents a critical determinant of fermented sausage safety, as the effectiveness of fermentation largely depends on the initial contamination level. Preventing *Salmonella* contamination in raw meat through effective hygienic practices and supply chain control is therefore essential.

Moreover, in the absence of microbiocidal steps in traditional SFS production, additional hurdles may be considered to further enhance safety. For instance, the product composition may influence pathogen persistence: the high fat content typical of fermented sausages can exert a protective effect on bacterial cells by limiting exposure to inhibitory factors [49,50]. Although not directly investigated in this study, the influence of fat content on *Salmonella* survival warrants further investigation. Similarly, acidification is widely recognized as the primary hurdle limiting pathogen growth in fermented sausages produced at temperatures above 20 °C, such as SFS [33]. In the present study, no fermentable carbohydrates were added, as the formulation was designed to reflect traditional SFS production, recognized as a traditional agri-food product. While carbohydrate supplementation may enhance acidification kinetics and influence pathogen behavior [58,59], its potential role in improving pathogen control in traditional SFS requires further investigation. Finally, given the absence of microbiocidal steps in traditional SFS production, post-process interventions may offer an additional safety margin without altering traditional formulation. Among these, high-pressure processing (HPP) has been shown to effectively reduce *Salmonella* in fermented meat products and is increasingly applied in the meat industry [56,60]. Further research and development are needed to refine these methods and ensure the feasibility of HPP in traditional SFS production.

## 5. Conclusions

The challenge test results highlighted the complex interaction of physicochemical and microbiological factors influencing the microbiological safety of Sardinian fermented sausages. Among the hurdles applied, acidification during early fermentation appeared to play a major role in reducing *Salmonella* levels, while a_w_ reduction and vacuum storage mainly contributed to growth inhibition rather than pathogen inactivation. Although a reduction in *Salmonella* was observed throughout fermentation and ripening, complete elimination was not achieved. These findings emphasize the critical importance of controlling the microbiological quality of raw materials, particularly in the absence of microbiocidal steps during production. Establishing clear guidelines for acceptable *Salmonella* levels in raw meat intended for fermented sausage production would represent a key preventive measure. In addition, incorporating additional hurdles could further improve the safety of the final product. Some limitations of the study should be acknowledged, including the use of pilot-scale production and artificial inoculation, which may not fully reflect industrial variability and natural contamination scenarios. In addition, although possible mechanisms and additional hurdles were discussed, these aspects were not directly investigated, and they warrant further targeted studies to further improve the understanding of *Salmonella* behavior in fermented sausages.

Overall, this study provides evidence-based insights into the factors governing *Salmonella* persistence in SFS and supports a comprehensive, hurdle-based approach to risk management. The results may assist producers in identifying critical control points along the supply chain and processing stages, contributing to improved safety and reliability of fermented sausage production.

## Figures and Tables

**Table 1:** pH and a_w_ values and microbiological counts in SFS control samples.

Time	pH	a_w_	*Salmonella*	ACC	ENT	CNS	LAB	YM
T_0_	5.89 ± 0.04	0.978 ± 0.01	<LOD	6.45 ± 0.34	2.69 ± 0.77	6.34 ± 0.13	3.85 ± 2.90	2.10 ± 0.10
T_1_	5.85 ± 0.04	0.974 ± 0.02	<LOD	6.41 ± 0.23	3.34 ± 1.04	6.12 ± 0.23	5.29 ± 0.48	2.98 ± 0.78
T_2_	5.59 ± 0.15	0.974 ± 0.02	<LOD	7.99 ± 0.55	4.29 ± 0.85	6.74 ± 0.55	7.74 ± 0.91	4.52 ± 0.45
T_7_	5.12 ± 0.14	0.950 ± 0.01	<LOD	7.51 ± 0.77	3.97 ± 1.19	6.89 ± 0.65	8.48 ± 0.34	4.34 ± 0.60
T_21_	5.52 ± 0.14	0.834 ± 0.02	<LOD	7.58 ± 0.59	3.15 ± 0.65	6.80 ± 0.81	8.34 ± 0.38	4.72 ± 0.53
T_30_	5.53 ± 0.23	0.840 ± 0.02	<LOD	7.74 ± 0.62	3.58 ± 0.74	7.02 ± 0.27	8.23 ± 0.19	3.97 ± 0.55
T_40_	5.69 ± 0.20	0.841 ± 0.02	<LOD	7.84 ± 1.04	3.02 ± 0.48	7.20 ± 0.33	8.30 ± 0.10	3.51 ± 0.37

ACC: aerobic colony count; ENT: *Enterobacteriaceae* count; CNS: coagulase-negative staphylococci; LAB: lactic acid bacteria; YM: yeasts and molds; <LOD: below limit of detection.

**Table 2:** pH and a_w_ mean values and microbiological counts in SFS artificially contaminated with *Salmonella* in the three batches.

Time	pH	a_w_	*Salmonella*	ACC	ENT	CNS	LAB	YM
T_0_	5.89 ± 0.09	0.978 ± 0.05	2.45 ± 0.33 ^ab^	6.33 ± 0.43	3.21 ± 0.65	6.38 ± 0.06	5.43 ± 0.48	2.47 ± 0.86
T_1_	5.87 ± 0.04	0.974 ± 0.02	2.33 ± 0.60 ^ab^	6.49 ± 0.21	3.48 ± 0.85	6.07 ± 0.20	5.33 ± 0.50	3.34 ± 0.28
T_2_	5.63 ± 0.14	0.973 ± 0.02	2.90 ± 0.69 ^a^	7.52 ± 0.41	4.54 ± 0.95	6.77 ± 0.57	7.50 ± 0.75	4.20 ± 0.10
T_7_	5.27 ± 0.35	0.948 ± 0.01	2.30 ± 0.85 ^b^	8.24 ± 0.21	4.24 ± 0.78	7.50 ± 0.42	8.54 ± 0.09	3.97 ± 0.57
T_21_	5.45 ± 0.18	0.814 ± 0.02	2.68 ± 0.43 ^ab^	7.79 ± 0.54	3.72 ± 1.01	7.32 ± 0.21	8.40 ± 0.12	4.28 ± 1.01
T_30_	5.54 ± 0.30	0.833 ± 0.01	2.50 ± 0.54 ^ab^	7.69 ± 0.59	3.95 ± 0.85	7.12 ± 0.32	8.33 ± 0.09	3.84 ± 0.79
T_40_	5.53 ± 0.15	0.828 ± 0.02	2.07 ± 0.80 ^b^	7.47 ± 0.41	3.50 ± 0.88	6.88 ± 0.20	8.30 ± 0.07	3.47 ± 0.89
T_100_	5.73 ± 0.29	0.823 ± 0.04	0.95 ± 0.85 ^c^	7.82 ± 0.83	2.07 ± 0.34	6.50 ± 0.58	8.24 ± 0.26	3.46 ± 0.22

ACC: aerobic colony count; ENT: *Enterobacteriaceae* count; CNS: coagulase-negative staphylococci; LAB: lactic acid bacteria; YM: yeasts and molds. Values are expressed as mean ± standard deviation. Different letters within the same column indicate statistically significant differences among sampling times (*p* < 0.05).

**Table 3:** Composition values (expressed as mean percentages) in SFS artificially contaminated with *Salmonella* in the three batches.

	Fats	NaCl	Proteins	Humidity
T_0_	29.29 ± 3.88	3.58 ± 0.05	18.83 ± 2.21	46.44 ± 2.28
T_2_	29.10 ± 5.56	3.59 ± 0.06	20.42 ± 0.97	45.84 ± 2.41
T_7_	36.23 ± 3.39	7.03 ± 9.25	19.97 ± 2.18	39.92 ± 1.78
T_21_	39.26 ± 11.12	4.92 ± 0.21	26.79 ± 7.46	28.16 ± 5.58
T_30_	47.79 ± 5.75	5.12 ± 0.30	21.07 ± 3.91	27.46 ± 2.50
T_40_	47.09 ± 9.18	4.57 ± 0.32	24.56 ± 6.06	23.44 ± 3.03

## Data Availability

The original contributions presented in this study are included in the article. Further inquiries can be directed to the corresponding author.
